# Why the study of comparative psychology is important to neuroscientists

**DOI:** 10.3389/fnbeh.2022.1095033

**Published:** 2023-01-10

**Authors:** Charles I. Abramson

**Affiliations:** Department of Psychology, Oklahoma State University, Stillwater, OK, United States

**Keywords:** comparative psychology, Morgan’s canon, systematic variation, neuroscience, definition

## Abstract

The purpose of this contribution is threefold. First, is to acquaint neuroscientists with the area of psychology known as comparative psychology. Comparative psychology is the oldest of the organized social sciences with the term appearing as early as 1808. Many of the myriad issues of experimental design routinely faced by comparative psychologists are directly applicable to neuroscience. These issues include consistent definitions of psychological phenomena, the use of Morgan’s canon to reduce unbridled anthropomorphism, and observation oriented modeling as a new statistical procedure to increase replication. Second, is a discussion of early comparative methods that may be of value to contemporary neuroscientists. Third, how the comparative approach can help the neuroscientist limit unfounded generalizations across species and develop more animal-friendly behavioral testing options tailored for the species or strain of interest. The articles closes with some recommendations on how comparative psychologists and neuroscientists can work more closely together.

## Introduction

I would like to thank Dr. Raffaele d’Isa for inviting me to share my opinion on the value of comparative psychology for neuroscientists. Comparative psychology (CP) is the application of the comparative method to problems in psychology (Abramson, 2018). CP is the oldest of the organized psychologies and arguable one of the first social sciences where researchers learned to make scientifically valid comparisons.

The issues of experimental design faced by comparative psychologists in its 215 year history are directly applicable to neuroscience. These issues include the importance of consistent definitions, the use of systematic variation as a control procedure, and an appreciation of Morgan’s canon to reduce unbridled anthropomorphism. I have discussed the importance of comparative psychology in several previous publications (e.g., Abramson, 1994, 1997, 2013, 2015, 2018; Abramson and Wells, 2018; Abramson and Levin, 2021).

## Brief history

The phrase “comparative psychology” appeared as early as 1808 in German (vergleichende psychologie) used by the physiological anthropologist Liebsch (1808), 1812 in Latin (psychologia comparata) used by the physician Hoffbauer (1812) and 1827 in Italian (psicologia comparata) used by the philosopher Poli (1827). In his book chapter *Of the science of comparative psychology. Origin, principles, critique, truthfulness and useful application of comparative psychology*, Poli (1827) defines CP as “the science that studies and analyzes the instincts, the functions and the habits of beasts in relation to the analogous human faculties, with the aim to explain better the phenomena of thought and feeling in man.^1^ “In 1836 the phrase was first used in French (psychologie comparée) by the physician Lélut (1836). Describing the field of observation of CP, Lélut mentions CP of animal species, of human races, of human ages and of mental pathologies. In English, the phrase was used in 1841 by psychiatrist and ethnologist Prichard (1841) and in 1858 by zoologist Weinland (1858). In 1864 Flourens (1864) published the first book with the phrase as its title: *Comparative Psychology* [*Psychologie comparée*, in the original French version].^2^ In 1876 Spencer (1876) published “*The comparative psychology of man*.” In 1880 Ludwig Büchner wrote, in his *“Mind in Animals,”* that “Comparative anatomy, i.e., the study of bodies, which we have long followed, must necessarily have beside it comparative psychology, the study of minds” (Büchner, 1880), and in 1882 George Romanes used the term “comparative psychology” in his “*Animal Intelligence*” (Romanes, 1882). Of particular interest, Alfred Binet, who developed intelligence tests that eventually became known as the Stanford-Binet Intelligence Quotient (IQ) test, published a book in 1889 on the “psychic life of micro-organisms” where he highlights the benefits of comparative psychology (Binet, 1889; Abramson and McCarthy, 2022).

The first CP society appeared in 1885 in Montreal, Canada: the Association for the Study of Comparative Psychology (Mills, 1887; Murray, 1990). In contrast, the American Psychological Association (APA) was founded in 1892 and the Society for Neuroscience was established in 1969—respectively, 7 and 84 years after the first CP Society.

Comparative psychology has always been identified with neuroscience. One only has to look at any CP textbook to appreciate that all contain at least one chapter related to the “physiology of behavior.” Moreover, in 1947 the APA created the *Journal of Comparative and Physiological Psychology*. This collaboration between comparative psychology and neuroscience was recognized until 1982, when the journal was split into the *Journal of Comparative Psychology* and *Behavioral Neuroscience*.

I will comment on several issues that I believe will be useful for neuroscientists. These are: inconsistent definitions, the use of systematic variation as a control procedure, the value of Morgan’s canon to limit anthropomorphizing, and the advantage of using observation orientated modeling to analyze data. I will also mention some early techniques that may be useful for contemporary neuroscience research and close with some recommendations.

## Inconsistent definitions

Neuroscience studies often deal with psychological and behavioral concepts. Unfortunately, many neuroscientists, especially those coming from a molecular background, overlook providing definitions for these concepts or take existing definitions for granted. This attitude is problematic and can lead to errors in both experimental design and data interpretation. Sometimes there is not a clear concept behind the term employed, so that the term is vague. In other cases, definitions for those concepts exist in the literature, but they are many and varied, so it is actually not possible to know which definition the authors of the study embrace.

Comparative psychology, on the other hand, being a branch of psychology, connects animal research with psychological theorizing. Hence, it could provide neuroscientists with the required theoretical support and help them develop objective definitions for psychological and behavioral concepts. When neuroscience studies use psychological concepts, clear definitions should always be provided, or at least, references to the scientific literature clarifying the theoretical background adopted by the authors.

The use of inconsistent definitions reduces the ability to replicate research and creates a situation where data obtained by neuroscientists may well rest upon an ever changing foundation of weak behavioral knowledge. If we are not more careful, psychology-related sciences and social science in general could become a discredit field much as Richard Feynman stated in a BBC interview in 1981 (Tavares, 2014).

One of the best examples of inconsistent definitions can be found in the study of learning. Neuroscientists may be surprised to discover that there are no consistent definitions of classical conditioning and operant conditioning (Abramson and Wells, 2018). Moreover, there are no consistent definitions of, for example, learning (Kimble, 1961; Bullock and Quarton, 1966), behavior (Levitis et al., 2009; Cvrčková et al., 2016), tool use (Crain et al., 2013), intelligence and personality (Sternberg, 1984; Sternberg and Detterman, 1986; Schlinger, 2003; Legg and Hutter, 2007). All of these areas are of interest to neuroscientists. How can a neuroscientist study a behavioral phenomenon when the definitions of that phenomenon is consistently shifting? The answer is you cannot.

In regards to intelligence, the intelligence of plants has become a popular area of neuroscience research (e.g., Abramson and Chicas-Mosier, 2016; Abramson and Calvo, 2018). How much faith can a neuroscientist have that they are investigating the “neuroscience of intelligence” (or learning, or tool use, or behavior, or personality) if there are no consistent definitions of what intelligence is? The answer is you cannot.

One of the most egregious examples is the definition of cognition. Frankly, I am not sure that anyone actually knows what “cognition” is. The founding editor of the journal *Cognition* was once asked to define it. The response was “Whatever I like” (Amsel, 1989). In one study, 12 leading cognitive textbooks were examined and 12 different definitions found (Abramson, 2013). How can a neuroscientist rationally study “cognition” if the term is so ambiguous?

Another issue is whether male/female differences among non-human animals should be referred to as gender differences or sex differences. I recently had the opportunity to review a paper on the exploratory behavior of male and female woodlice where the authors referred to sex differences as “gender differences.” While I found the notion of gender differences in woodlice, or any non-human animal problematic, it nicely illustrates how psychological concepts developed for humans (such as personality) are seeping into the natural science community to the determent of the science. A definition of what distinguishes gender from biological sex, and a comparative analysis directed toward understanding if, and in which non-human animals gender can be present, would be most welcome.

## Systematic variation

In addition to definitional problems, the neuroscientist should be aware of what is known in the CP literature as “systematic variation.” Systematic variation is a control procedure where the experimenter “systematically varies” possible explanations before reaching a conclusion (Abramson, 1994). Systematic variation is a reminder to neuroscientists that alternative explanations must be evaluated before inferring that, for example, a species, subspecies, strain or sex difference actually exists.

Consider, for instance, a human study in which females outperform males on an intelligence test. Setting aside problems with the definition of intelligence, neuroscientists not familiar with comparative research methods might conclude that “females are more intelligent than males.” While this may be correct, it cannot be concluded before possible explanations are “systematically varied.” Males may not be motivated to complete the task. Therefore, motivation will have to be systematically varied and if the differences among males and females persist, then motivation is ruled out. Once motivation is ruled out, the researcher may direct attention to the properties of the intelligence test. Perhaps the test itself contains some inherent methodological bias favoring females. If, using a methodologically different test assessing the same type of intelligence, females still outperform males, then the researcher may be confident that a difference between the sexes exists for this particular task. While the above example focuses on humans, the logic of systematic variation is exactly the same when considering experimental designs with non-human animals.

## Morgan’s canon

Systematic variation is a control method that limits unsupported generalizations related to comparative research. Another comparative strategy useful for neuroscientists is known as Morgan’s canon. Morgan’s canon is an epistemological position that encourages researchers to limit their speculations when making comparisons (Karin-D’Arcu, 2005).

The original statement of the canon appeared in Conwy Lloyd Morgan’s *Introduction to comparative psychology* (Morgan, 1894). As the original statement was often misunderstood, he clarified the canon in a later publication (Morgan, 1903). As Morgan states (1903, page 59):

“In no case is an animal activity to be interpreted in terms of higher psychological processes, if it can be fairly interpreted in terms of processes which stand lower in the scale of psychological evolution and development. To this, however, it should be added, lest the range of the principle be misunderstood, that the canon by no means excludes the interpretation of a particular activity in terms of the higher processes, if we already have independent evidence of the occurrence of these higher processes in the animal under observation.”

The canon contains several important principles for neuroscience research. First, researchers must not assume a higher level of processing if a lower level can satisfactory account for the data. Secondly, one must view with caution the tendency to anthropomorphize human explanations of behavioral phenomena to non-human animals. Third, a researcher must not overlook the possibility that a more reasonable and fundamental explanation of a non-human animal’s behavior may be appropriate also when observing the same behavior in humans.

## Statistical analysis—Observation oriented modeling

A difficult challenge facing neuroscience researchers is what statistics to use. I suggest neuroscientists consider Observation Oriented Modeling (OOM) (Grice, 2011; Grice et al., 2012). OOM is a collection of methods requiring researchers to hypothesize an expected pattern of results and then determine how many individuals or entities match that predicted pattern (Grice, 2021). OOM has been used in a number of investigations including social reinforcement delays (Craig et al., 2012), timing (Craig et al., 2014, 2015), and taste aversion learning (Varnon et al., 2018). The program is easy to use and well supported. While I know of no specific case where OOM has been used in neuroscience research, I believe it is worth looking into.

## Importance of resisting reductionism

Natural sciences place an emphasis on reductionism. This is easily seen in fields such as chemistry, genetics, molecular biology and indeed neuroscience where their traditions favor experimental designs that focus on internal validity and reducing variability due to external factors (i.e., the factors different from the experimental factor or factors of interest). This type of variability is defined as “noise” and considered a possible source of uncontrolled error in the experiment. Internal validity evaluates if the experimental design, conduct and data analysis answer the experimental questions of a study without bias, whereas external validity refers to the extent to which the experimental finding can be generalized to a different contexts (Andrade, 2018). Research in CP has consistently shown that while internal validity is important, it should not be at the expense of external validity (Steckler and McLeroy, 2008).

While the reduction of noise in experimental design is important, the neuroscientist should remember that human and non-human animals live in a world of noise. Mice, and other rodent models so favored in behavioral neuroscience, live in a world of constantly changing environmental conditions including temperature and humidity fluctuations, and exposure to stressors such as pesticides and pollutants, all of which influences development across the life span. There is a real danger that the reductionist models do not represent external validity as non-human animal models often are studied, maintained, and created in temperature-controlled, humidity-controlled and specific pathogen free (SPF) environments with little contact with outside environmental influences—i.e., noise. In my opinion, one method to ensure external validity is to incorporate systematic variation into the experimental designs used by neuroscientists. At the very least, there should be some recognition by neuroscientists that the reductionist models may not represent the entire picture, could be misleading, and could represent a disciplinary standard detrimental to the quality of the science.

## Comparative methods to investigate rodent behavior

I suggest neuroscientists examine some of the early to mid-20th century research methods developed by comparative psychologists. Many of these methods are no longer in use and, in my view, just waiting to be rediscovered and adapted for contemporary research. Of particular interest to neuroscientists is that they were designed specifically to investigate human phenomena in non-human animals from a comparative perspective.

One of the most interesting is the work of Walter Samuel Hunter on the delayed reaction in animals and children (Hunter, 1913). The monograph describes a learning task where the subject must delay its response before a reward is obtained. This task has been used to compare the performance of children, rats, dogs, and raccoons. Many other tasks can be found in Norman Leslie Munn’s *Handbook of psychological research on the rat* (Munn, 1950). There is literally page after page of fascinating material including experimental designs related to what is considered “cognitive” such as reasoning and the use of logic. Another excellent source is the three volume set on comparative psychology by Carl John Warden, Thomas Nichols Jenkins, and Lucien Haynes Warner (Warden et al., 1935, 1936, 1940). Once again, a fascinating array of methods and experimental paradigms are presented.

Why are these techniques not generally known? I believe it is the lack of interest in history generally, and of the history of psychology in particular. Professors of neuroscience probably do not realize that before the introduction of simple mazes and runways, comparative psychologists of the first decades of 20th century confronted their organisms with an array of sophisticated problems. These problems include multiple unit mazes, elevated mazes, temporal mazes, jumping stands, and a variety of situations in which the organism must escape an enclosure by solving a puzzle (Munn, 1950). Many of these techniques were designed to explore what are now considered “cognitive” processes. However, and this is often overlooked, processes such as learning and insight were then studied and interpreted within a behaviorist but not a cognitivist framework (Abramson, 2018; Abramson and Levin, 2021).

Importantly, I would like to note that research performed by comparative psychologists during its golden age used a variety of organisms. As time progressed, the range of organisms became restricted to mostly rats and pigeons, as did the type of apparatuses used—a situation similar to what is facing the contemporary neuroscientist, which employs mainly mice and rats.

Such a situation should serve as a warning to neuroscientists that it is dangerous to rely on a single or even a few species to base conclusions on. For example, there are 38 species of mouse and they differ in many respects related to sensory abilities, natural history and behavior. Nevertheless, in neuroscience *Mus musculus* is generally considered the standard mouse. Generalizing findings from a single species to an entire genus is fraught with difficulty and wrong generalizations can easily be made. Analogously, the mouse strain C57BL/6 is often considered the standard strain, leading to a widespread bias in the choice of the experimental subjects (Zilkha et al., 2016).

## Discussion

Problems with definitions, anthropomorphism, and difficulties with replication, are all problems addressed by CP. Arguably, the most important contribution that CP can give to neuroscience is the “comparative” approach itself.

Neuroscientists often perform experiments on a single model and may believe that their discoveries are universally valid. The results of a memory study of mice, for example, are considered to be valid for “memory” in general, not for “mouse memory.” Many neuroscience investigations have a strong translational goal and what is found in a model organism is implicitly considered to be related to what happens in humans. Taking this relationship for granted is very dangerous. A major reason why treatments that are found to work in model non-human animals often do not work when applied to humans, is because species-specific differences are present and not appreciated until it is too late.

Comparative psychology, on the other hand, emphasizes that each species has its own specific natural history, behavioral tendencies, learning practices and neural processes. Thus, a model developed with one species should be tested also with closely related species within a family or even closely related strains within a same species. Only in this way can generalizations among models be safely made. When differences are found, since the genetic and neural organization between the experimental subjects is so similar, it would be much easier, by subtraction, to identify the genetic and neural substrates of the observed difference between the species or the strains. Such a comparative approach would be very useful in neuroscience research to identify, by contrast, the neurobiological underpinnings of behavior.

Practically speaking, behavioral neuroscientists should try to assess “cognitive” and behavioral function in multiple species. Since rodents are the most popular model organism in neuroscience, the same task could be tested, for example, in mouse, rat, hamster, and gerbil. If, more specifically, mice are used, then experiments should not be limited to the use of one single strain, but the discovery should be reconfirmed (or disproved) by testing at least three or four different strains. Sex differences should also be taken into account. Too often in neuroscience, and generally in biomedical sciences, experiments are performed on only one sex (generally male) and results have been generalized as universally valid. Experiments on females could lead to completely different results. Indeed, a more frequent inclusion of females in neuroscience studies would avoid inappropriate generalizations deriving from a sex bias (Prendergast et al., 2014; Zilkha et al., 2016).

A comparative approach would require a higher number of experimental subjects. Nonetheless, it would help to ameliorate the reproducibility crisis that biological sciences are currently facing. In the end, obtaining solid results could actually lead to a reduction in the total number of experimental animals used, since a lower number of independent studies would be required to reconfirm the results. Furthermore, even if multiple sexes, strains or species are not used in the same study, the important point would be at least to adopt the comparative approach as a *forma mentis*, to avoid inaccurate generalizations. If only one of many options can be employed in a study, for instance a single sex or a single strain, a rationale for that choice should be provided, based on general knowledge of biological processes or on previous experimental data.

Comparative psychology could be helpful in avoiding failure of behavioral experiments and useless employment of animals. For instance, a recent good example of adoption of a comparative approach is a study by König et al. (2020) in which voluntary physical activity and energy expenditure were measured in both sexes of 30 strains of mice, recording the parameters in both the light phase (photophase) and dark phase (scotophase) of the day. Interestingly, the study found that not all strains, and within some strains not both sexes, had light-dark cycles. If an experiment on circadian rhythms of physical activity has to be performed, choice of a strain with no light-dark variation would lead to failure of the experiment. A comparative knowledge of the different strain and sex characteristics can lead to the choice of the most suitable model for the target behavior, reducing the number of failed experiments and hence the total number of animals needed to obtain a valid result.

Comparative psychology can also help neuroscientists develop more animal-friendly behavioral testing options tailored for the species or strain of interest. For instance, in a recent CP study, the palatability of over 30 types of food was assessed in rats and significant differences among rat strains were found regarding food type preference (Dews et al., 2022). Such comparative data may help neuroscientists choose the best food reward in appetitively motivated learning tests, optimizing training and avoiding the necessity of food deprivation to motivate the animals.

A consideration of CP will also encourage the behavioral neuroscientist to have at least a working knowledge of their model’s natural and evolutionary history. Where does their model organism live? Does it invade diverse environments or is it restricted to a narrow niche? Does it eat meat, plants, or both? Does the model organism live alone or in groups? Only with such information (i.e., noise), and acting upon it, can the behavioral neuroscientist ensure that their models make contact with the natural environment.

Finally, I would like to offer some recommendations. First, behavioral neuroscientists should acquaint themselves with CP. As I have endeavored to show in this opinion article, CP has much to recommend it for behavioral neuroscience in terms of both research design and general overall strategy. Second, I would strongly encourage behavioral neuroscientists to collaborate with comparative psychologists in the design and interpretation of their experiments.

For readers interested in obtaining source material about comparative psychology, see Abramson (2018). This article contains information related to review articles, textbooks, history, and recommended papers. It was part of a special issue on comparative psychology appearing in the *International Journal of Comparative Psychology*. The remaining 12 articles in that special issue focus on methodology, applied aspects, and teaching, respectively (Abramson and Hill, 2018). In addition, there is a companion issue solely dedicated to the teaching of CP (Abramson, 2020). The 12 articles in that special issue describes over 50 inquiry-based activities. Both special issues can be downloaded free of charge.

## Author contributions

The author confirms being the sole contributor of this work and has approved it for publication.

## Footnotes

1 Title and quotation translated from the original Italian by Dr. Raffaele d’Isa.

2 Flourens had previously used the phrase comparative psychology in 1861 in his book *De la raison du génie et de la folie*, in which he dedicated to the topic a whole chapter entitled *De la psychologie comparée et du sense intime* (Flourens, 1861).
